# GSK3β-Dependent Phosphorylation Alters DNA Binding, Transactivity and Half-Life of the Transcription Factor USF2

**DOI:** 10.1371/journal.pone.0107914

**Published:** 2014-09-19

**Authors:** Tina Horbach, Tabughang Franklin Chi, Claudia Götz, Satyan Sharma, André H. Juffer, Elitsa Y. Dimova, Thomas Kietzmann

**Affiliations:** 1 Faculty of Biochemistry and Molecular Medicine, Biocenter Oulu, University of Oulu, Oulu, Finland; 2 Department of Chemistry, University of Kaiserslautern, Kaiserslautern, Germany; 3 Medical Biochemistry and Molecular Biology, Saarland University, Homburg, Germany; North Carolina State University, United States of America

## Abstract

The upstream stimulatory factor 2 (USF2) is a regulator of important cellular processes and is supposed to have also a role during tumor development. However, the knowledge about the mechanisms that control the function of USF2 is limited. The data of the current study show that USF2 function is regulated by phosphorylation and identified GSK3β as an USF2-phosphorylating kinase. The phosphorylation sites within USF2 could be mapped to serine 155 and threonine 230. In silico analyses of the 3-dimensional structure revealed that phosphorylation of USF2 by GSK3β converts it to a more open conformation which may influence transactivity, DNA binding and target gene expression. Indeed, experiments with GSK-3β-deficient cells revealed that USF2 transactivity, DNA binding and target gene expression were reduced upon lack of GSK3β. Further, experiments with USF2 variants mimicking GSK3β phosphorylated USF2 in GSK3β-deficient cells showed that phosphorylation of USF2 by GSK3β did not affect cell proliferation but increased cell migration. Together, this study reports a new mechanism by which USF2 may contribute to cancerogenesis.

## Introduction

The upstream stimulatory factors (USFs) are involved in the transcriptional regulation of various genes whose products contribute to the stress and immune response, to cell cycle and proliferation as well as to lipid and carbohydrate metabolism [1]. In mammals, two different *usf* genes were identified. The *usf1* and *usf2* genes are ubiquitously expressed with varying ratios in different organs [2]. In addition, alternative splicing of the USF2 pre-mRNA gives rise to the generation of USF2a and USF2b [3]. All USF proteins belong to the basic helix-loop-helix leucine zipper (b-HLH-LZ) transcription factor family [4]. They contain a highly conserved USF-specific region (USR) [5] and bind preferably as USF1/USF2 heterodimers [3] to E-boxes with a 5′-CANNTG-3′ core sequence in the promoter of their viral or cellular target genes [6].

Generation and observation of USF knockout mice revealed that USF2 seems to be the more important USF variant. While USF1^−/−^ mice exhibited a rather normal phenotype, USF2^−/−^ mice had a reduced lifespan, decreased fertility and were 20–40% smaller than their wild-type litter mates [7]. Furthermore, USF2 seems to be linked to the development of cancer. While several studies indicated a tumor-suppressive function of USF2 in prostate cancer [8], breast cancer [9] and oral cancer [10], a pro-proliferative function of USF2 was observed in lung cancer cells [11].

Although the above-mentioned studies indicate that USF2 has a crucial role in the organism, especially during cancerogenesis, there is not much knowledge about the mechanisms regulating the activating or suppressive functions of USF2. Eukaryotic cells feature numerous different mechanisms for the regulation of transcription factor function. One versatile option to reversibly regulate the activity of transcription factors is protein phosphorylation. While there is some evidence that USF1 can be phosphorylated by various kinases participating in different signaling pathways [12]–[20], studies indicating USF2 phosphorylation are rather limited and only one identified PKA as a USF2-phosphorylating kinase [17].

Our own previous work on the role of USF2 on the expression of the cancer marker plasminogen activator inhibitor-1 (PAI-1) [21] suggested that USF2 might be phosphorylated by other kinases then PKA. A kinase which might be of special interest in this regard is glycogen synthase kinase 3β (GSK3β). This kinase links the PI3K-PKB (AKT) pathway and the Wnt/β-catenin signaling pathway which are both regulating various cellular processes including embryonic development, protein synthesis, cell proliferation, cell differentiation and cell motility [22]–[24]. GSK3β is an unusual kinase since it is active under resting conditions and is inhibited both by a PKB (AKT) activating growth factor or a Wnt signal [25]. GSK3β appears to be an attractive candidate kinase for the regulation of USF2 since both proteins seem to be involved in the development of different types of cancer, especially prostate cancer [8], [26], [27]. In addition, we found that parts of the USF2 protein sequence match with the substrate recognition preferences of GSK3β [28]. Therefore, it was the aim of the present study to analyze the impact of GSK3 on the phosphorylation and function of USF2.

We demonstrate that USF2 can be phosphorylated by GSK3β at two distinct sites. Our data show that these phosphorylation events have the ability to regulate the function of USF2 by altering the DNA binding capacity, the transactivity and the protein stability. Furthermore, we demonstrate that cell migration is influenced by GSK3β-mediated phosphorylation of USF2.

## Materials and Methods

### Materials

All biochemicals and enzymes were of analytical grade and purchased from commercial suppliers. As GSK3 inhibitors, LiCl (Sigma-Aldrich), 6-bromoindirubin-3-oxime (BIO), and 1-Azakenpaullone (both Enzo Life Sciences) were used in the concentrations indicated. BIO and 1-Azakenpaullone were dissolved in DMSO, LiCl in water.

### Plasmid constructs

The expression vector for FLAG-myc-tagged wild-type USF2a (USF2-WT) was generated by PCR using the forward primer 5′-CTGAGAAGCTTATGCTGGACCCGGG-3′ and reverse primer 5′-CTGAGTCTAGACTGCCGGGTGCCC-3′ and a previously described USF2a plasmid [29] as a template. The resulting PCR product was then cloned into the HindIII and XbaI sites of p3xFLAG-myc-CMV24 (Sigma-Aldrich). The forward primer 5′-CAGGGAATTCATGGACATGCTGGAC-3′ and the reverse primer 5′-GTATACTCGAGCTGCCGGGTGCCC-3′ were used to clone a wild-type USF2 product into the EcoRI and XhoI sites of the pGEX-5×1 plasmid (GE Healthcare). The plasmid pcDNA6A-Gal4 containing the Gal4 DNA binding domain was generated by cutting the DNA encoding the Gal4 DNA binding domain from the pSG424 vector [30] and ligating it into the pcDNA6A-Myc-His vector (Life Technologies) via the HindIII and BamHI restriction sites. The plasmid pcDNA6A-Gal4-USF2, allowing expression of a fusion protein of the Gal4 DNA binding domain and the N-terminal part of USF2, was generated by cloning a PCR fragment encoding the N-terminal part of USF2 into the BamHI and XbaI sites of pcDNA6a-Gal4; for the PCR the forward primer 5′-GTATGGTACCTAGGATCCATATGGACATGCTGGACCCGGGTCTCGATCCCGCTGCCTC-3′ and the reverse primer 5′-CGCGTCTAGAGAATTCCTAGGGTGTTCTGG TTCCATC-3′ were used. For the generation of deletion and point mutants, the QuikChange Site-Directed Mutagenesis Kit (Agilent Technologies) was used. The expression vectors for pcDNA3-GSK3β-WT, -S9A and -K85A were described before [31], [32] and obtained from Addgene (#14753, 14754 and 14755). The pFR-5Gal4-RE-Luc plasmid and the pRL-SV40 plasmid were purchased from commercial suppliers (Agilent Technologies and Promega, respectively) and the luciferase gene constructs containing the promoter regions of human HO-1 from −4000 to +80 [33], of human PAI-1 from −806 to +19 [34] and of rat FAS from −220 to +25 [35] have been described.

### Cell culture, transient transfections and luciferase assays

HeLa and HepG2 cells were maintained in Earle’s minimum essential medium (MEM, Sigma-Aldrich); GSK3β^+/+^ and GSK3β^−/−^ mouse embryonic fibroblasts (MEFs), a kind gift from J. Woodgett, were cultured in Dulbeccós modified Eaglés medium (DMEM, Sigma-Aldrich). These immortalized MEFs were derived according to approved protocols from GSK3β nullizygous embryos as described [36]. All media were supplemented with 10% fetal bovine serum (FBS, Biochrom), 1% non-essential amino acids and 0.5% ciprofloxacin. The cells were culture at 37°C in an atmosphere of 16% O_2_, 5% CO_2_, 79% N_2_ and 97% humidity. Transient transfections were conducted by the calcium phosphate precipitation method [37] or by using the **Amaxa MEF 1 Nucleofector** Kit (VPD-1004, Lonza).

For luciferase assays the transfection efficiency was controlled by cotransfection of a Renilla luciferase expression vector. In brief, one day before transfection 2×10^5^ HeLa cells were seeded in 2.5 ml growth medium in 60 mm cell culture dishes. The cells were transfected overnight with 3 µg of a Firefly luciferase gene construct, 1 µg of expression vector and 50 ng of the Renilla luciferase reporter plasmid (pRL-SV40). The next morning, the medium was replaced by fresh growth medium and the cells were cultured for further 26 h. Then luciferase assays were performed as previously described [38], [39].

### RNA preparation and quantitative real-time PCR

Isolation of total RNA was performed using the Qiagen RNeasy Mini Kit (Qiagen, Switzerland) following the manufacturer’s instructions. One µg of total RNA was used for first strand cDNA synthesis with the iScript cDNA synthesis Kit (Bio-Rad, Finland). Quantitative real-time PCR was performed in an **Applied Biosystems 7500** Real-Time PCR System (Life Technologies, Finland) by using an Applied Biosystem Power SYBR green PCR master mix (Life Technologies, Finland). The following primers were used: USF2 forward 5′-GCGTTCGGCGACCACAATA-3′, USF2 reverse 5′-GACTACGCGGTATGTCACCTG-3′, FAS forward 5′-GGAGGTGGTGATAGCCGGTAT-3′, FAS reverse 5′-TGGGTAATCCATAGAGCCCAG-3′, HO-1 forward 5′-GATAGAGCGCAACAAGCAGAA-3′, HO-1 reverse 5′-CAGTGAGGCCCATACCAGAAG-3′, PAI-1 forward 5′-GTGAATGCCCTCTACTTCAGTG-3′, PAI-1 reverse 5′-GCTGCCATCAGACTTGTGGAA-3′, ActB forward 5′-ATGCTCCCCGGGCTGTAT-3′, ActB reverse 5′-CATAGGAGTCCTTCTGACCCATTC-3′, Hprt forward 5′-CGAAGTGTTGGATACAGGCC-3′, Hprt reverse 5′-GGCAACATCAACAGGACTCC-3′. All primer sets were validated for their product and amplification efficiency using standard dilution analysis and melting curve analysis. β-Actin and Hprt (Hypoxanthine guanine phosphoribosyltransferase) were used as internal controls to normalize the variability in expression levels. The experiments for each data point were carried out in triplicate. The relative quantification of gene expression was determined using the ΔΔCt method [40].

### Western blotting, co-immunoprecipitations, protein phosphorylation and half-life studies

Cells were washed with cold PBS and scraped in lysis buffer (50 mM Tris pH 8.0, 5 mM EDTA, 150 mM NaCl, 5 mM DTT, complete mini protease inhibitors (Roche), 25 mM NaF, 1 mM Na_3_VO_4_, 25 mM β-glycerophosphate). The cells were then treated for 20 min in an ice-cooled ultrasonic water bath and subsequently centrifuged at 4°C and 13700×g for 20 min. The protein concentration was estimated by the Bradford method.

For the analysis of proteins by Western blotting, cell extracts were denaturated with 4x SDS sample buffer (500 mM Tris pH 6.8, 30% glycerol, 10% SDS, 0.01% bromophenol blue, 40 mM DTT) by incubating at 95°C for 10 min. Then the proteins were resolved by 10% SDS-PAGE and blotted to a nitrocellulose membrane. For the analysis of p-USF2, an acrylamide:bisacrylamide ratio of 200∶1 was used in the resolving gel.

As primary antibodies monoclonal antibodies against USF2 (6A9; 1∶1000; Cat.Nr. H00007392-M03, Abnova), HA-tag (F-7; 1∶500; Cat.Nr. sc-7392, Santa Cruz Biotechnology), myc-tag (9B11; 1∶1000; Cat.Nr. 2276, Cell Signaling Technology) and α-tubulin (B-5-1-2; 1∶10000; Cat.Nr. T6074, Sigma-Aldrich), as well as polyclonal antibodies against USF2 (N-18; 1∶500; Cat.Nr. sc-861, Santa Cruz Biotechnology) were used. Horseradish peroxidase (HRP)-conjugated goat anti-mouse (1∶5000, Cat.Nr. 170-6516, Bio-Rad) and goat anti-rabbit IgGs (1∶5000; Cat.Nr. 170-6515, Bio-Rad) were used. The enhanced chemiluminescence (ECL) system (GE Healthcare) was used for detection.

For immunoprecipitation experiments, protein samples from HeLa, GSK3β^+/+^, and GSK3β^−/−^ cells were prepared in lysis buffer (50 mM Tris/HCL, pH 7.5, 150 mM NaCl, 1% Triton X-100, 2 mM EDTA, 2 mM EGTA, 1 mM PMSF and complete protease inhibitor cocktail tablet (Roche)). After scraping, lysates were incubated with continuous shaking at 4°C for 20 min and then they were centrifuged at 12.000 g at 4°C for 15 min [41]. To recover USF2 immunoprecipitates, 250 µg of total protein was incubated with 2 µg antibody for 1 h at 4°C before Sepharose beads (30 µl per reaction mixture) were added for 12 h. Thereafter, the beads were washed five times with lysis buffer and recovered, pellets were dissolved in 2 X Laemmli buffer, loaded onto a SDS-PAGE gel, blotted and detected with antibodies against phospho-serine and phospho-threonine, respectively.

For dephosphorylation experiments, cell extract (90 µg protein) was incubated with 40 u calf intestinal phosphatase (CIP, Finnzymes) at 37°C for 5 min. In the appropriate control sample the enzyme was replaced by water.

In half-life studies, GSK3β^+/+^, and GSK3β^−/−^ cells were treated with 10 µg/ml cycloheximide (CHX, Sigma- Aldrich) for the indicated time periods. Whole cell extracts were prepared as above and 100 µg of protein was loaded onto 10% SDS- polyacrylamide gel. After electrophoresis and electroblotting onto a nitrocellulose membrane, proteins were detected with antibodies against USF2 and quantified with Fiji (NCBI) software.

### Phosphoprotein staining

Immunoprecipitated proteins (250 µg endogenous proteins or 150 µg overexpressed proteins) were denaturated and subjected to 10% SDS-PAGE. Phosphoproteins were stained with Pro-Q Diamond Phosphoprotein Stain (Life Technologies) and detected with a wavelength of 532 nm using the Typhoon 9400 (GE Healthcare). Subsequently, total proteins were detected by silver staining [42].

### Expression and purification of proteins, radioactive kinase assays

Using the pGEX-5×1 system (GE Healthcare), GST fusion proteins were expressed in *E. coli* BL21 (DE3) at 30°C and purified with Glutathione Sepharose beads as described previously [43]. Purified GST fusion proteins (1.5 µg) or proteins immunoprecipitated from whole cell extracts (350 µg) were incubated in kinase assay buffer (15 mM MOPS pH 7.2, 15 mM MgCl_2_, 3 mM EGTA, 1.2 mM EDTA, 150 µM DTT, 6 mM β-glycerophosphate) containing 20 ng of active, recombinant full-length human GSK3β (SignalChem), 50 µM unlabeled ATP and 1.5 µCi [γ^−32^P] ATP at 30°C for 20 min. The proteins were denaturated by incubating with 4x SDS sample buffer at 95°C for 10 min. Following separation of the proteins by 10% SDS-PAGE, the gel was exposed to a Kodak phosphor imaging screen overnight and thereafter autoradiography was detected with a Molecular Imager FX using the Quantity One software (all BIO-RAD). Afterwards, the total proteins were detected by Coomassie staining or silver staining.

### Chromatin immunoprecipitation (ChIP)

Wild-type (WT) and GSK3β^−/−^ MEFs were seeded in 15 cm cell culture dishes and ChIP was performed as described previously [44]. In brief, after cross-linking, cell lysis and sonication, chromatin samples were incubated with anti-USF2 antibody (N-18, Cat.Nr. sc-861, Santa Cruz Biotechnology), anti-RNA polymerase II antibody (N-20, Cat.Nr. sc-899X, Santa Cruz Biotechnology) or IgG preimmune serum (BioScience) in an ultrasonicator bath for 1 h at 4°C; after centrifugation, the precleared samples were mixed with protein G Sepharose beads for 1 h at 4°C. After several washes, Chelex 100 (Bio-Rad) suspension was added to the beads and the samples were boiled for 10 min; after cooling down to RT, Proteinase K was added and the samples were incubated for 30 min at 55°C with shaking, boiled again for 10 min and centrifuged [44]. The isolated DNA was subjected to quantitative PCR that was performed with the 7500 Real-Time PCR System (Applied Biosystems) using the Power SYBR Green Master Mix (Life Technologies). The ChIP primers used were: PAI-1 forward, 5′-GTCTAGACGACCGACCAGCCAAA-3′, PAI-1 reverse, 5′-GAAATGTCTGGGCTGCCCGC-3′. HO-1 forward, 5′-GCCTCCGGGCTGGATGTTG-3′ and HO-1 reverse, 5′-GGAGACCGTGAGCGAGCAGC-3′, FAS forward, 5′-GACGCTCATTGGCCTGGGC-3′ and FAS reverse, 5′-ACGCCGCTGCCGTCTCTCT-3′. As a positive control, a RNA polymerase II antibody was used to immunoprecipitate RNA polymerase II from an actively expressed housekeeping gene promoter; the mouse β-actin promoter was amplified with the following forward 5′-GTGAGTGAGCGACGCGGAGCCAA-3′, and reverse 5′-CTTACCTGGTGGCGGGTGTGGA-3′ primers [45]. All primer sets were validated for their product and amplification efficiency using standard dilution analysis and melting curve analysis. Differences in the USF2 and RNA Polymerase II DNA binding efficiency in wild-type (WT) and GSK3β^−/−^ MEFs were calculated by the percent input method using the formula triplicate average Ct100*2∧(Adjusted input - Ct (IP)), where input adjustment is calculated using the formula raw Ct(Ct Input-log2 of the dilution factor) (dilution factor (DF) is equal to 1/fraction input).

### Proliferation and cell migration assay

For BrdU, MTT and cell migration assays GSK3β^−/−^ MEF cells were seeded onto 10 cm cell culture dishes and one day later the cells were transfected by using the **Amaxa MEF 1 Nucleofector** Kit (VPD-1004, Lonza) with 5 µg of expression vectors for USF2-S155A/T230A, USF2-S155D/T230D or an empty vector. After 20 h, 4×10^4^ electroporated cells were seeded in 500 µl growth medium into a 24-well culture dish for the MTT assay, 1×10^4^ electroporated cells were seeded in 100 µl growth medium into a 96 well culture dish for the BrdU assay and 1×10^4^ electoporated cells were seeded for migration assay (see below). From another aliquot of the electroporated MEFs whole cell extracts were prepared for the control of transfection efficiency by Western blotting. Cells plated for the MTT assay and the BrdU assay were cultured for further 30 h. For the MTT assay cells were stained with 50 µl MTT solution (4 mg/ml in PBS) for 1.5 h and the absorbance was measured at a wavelength of 570 nm. The BrdU assay (Calbiochem) was performed according to the manufacturer’s instructions and the absorbance was measured at dual wavelengths of 450–540 nm.

Cell migration was measured by using medium-treated 24-well Transwell chambers (BD Bioscience) with 8.0 µm polycarbonate membranes. The bottom chamber was filled with 600 µl medium containing 10% fetal calf serum; cells were seeded into the top chamber at a density of 1×10^4^ cells per well in 100 µl serum-free medium. After incubation in a humidified incubator with 5% CO_2_ at 37°C for 4 h, cells were fixed with 4% paraformaldehyde and the non-migratory cells were scraped off from the top of the Transwell with a cotton swab. The migrated cells attached to the bottom chamber were stained with 0.0075% crystal violet. Crystal violet absorption at 595 nm was measured in a Tecan Infinite M1000Pro microplate reader. For each migratory condition, two identical replicates were performed.

### In silico analysis of phosphorylated USF2 structures

A homology model of USF2 was built using the I-TASSER online software [46], [47], followed by an energy minimization of the predicted structure. The energy minimization and subsequent computer simulations were all completed with Gromacs 4.0.7. [48] and GROMOS96 43a1 force field [49]. The residues S155 or T230 were phosphorylated employing the molefacture plugin in VMD [50].

The resulting structures (native, S155 phosphorylated, T230 phosphorylated) were each solvated in a rectangular box containing SPC waters [51], under periodic boundary conditions. After a steepest-descent energy minimization of each system until they converged to 10 kJ mol^−1^ nm^−1^, a short 100 ps in the NVT (constant number of atoms, constant volume, constant temperature) ensemble was carried to equilibrate the given system. This was followed by a 15 ns production run in the NPT (constant number of atoms, constant pressure, constant temperature) ensemble. All simulations were carried out at 1.0 atm constant pressure using the Parrinello-Rahman barostat [52] and at 300 K constant temperature using V-rescale algorithm [53] with coupling constants of 0.5 and 0.1 ps, respectively. The time step used for the simulations was 2 fs. A cut-off of 12 Å was employed for the short-range non-bonded interactions. Long-range electrostatic interactions were computed using the particle mesh Ewald (PME) method [54]. All bonds were constrained employing the LINCS algorithm [52]. Water molecules were constrained using the SETTLE algorithm [55].

### Statistical analysis

Each experiment was performed at least three times and representative data are shown. Data in bar graphs are given as mean values ± standard deviation (SD). Statistical differences were calculated by using the Student t-test with error probabilities of p<0.05 to be significant.

### Ethics statement

All experimental procedures involving animal-derived cell lines were approved and performed only under permissions from the Animal Experiment Board of Finland following the regulations of the EU Directive 86/609/EEC, the European Convention ETS123 and the national legislation of Finland.

## Results

### GSK3β phosphorylates USF2

**Figure 1:**
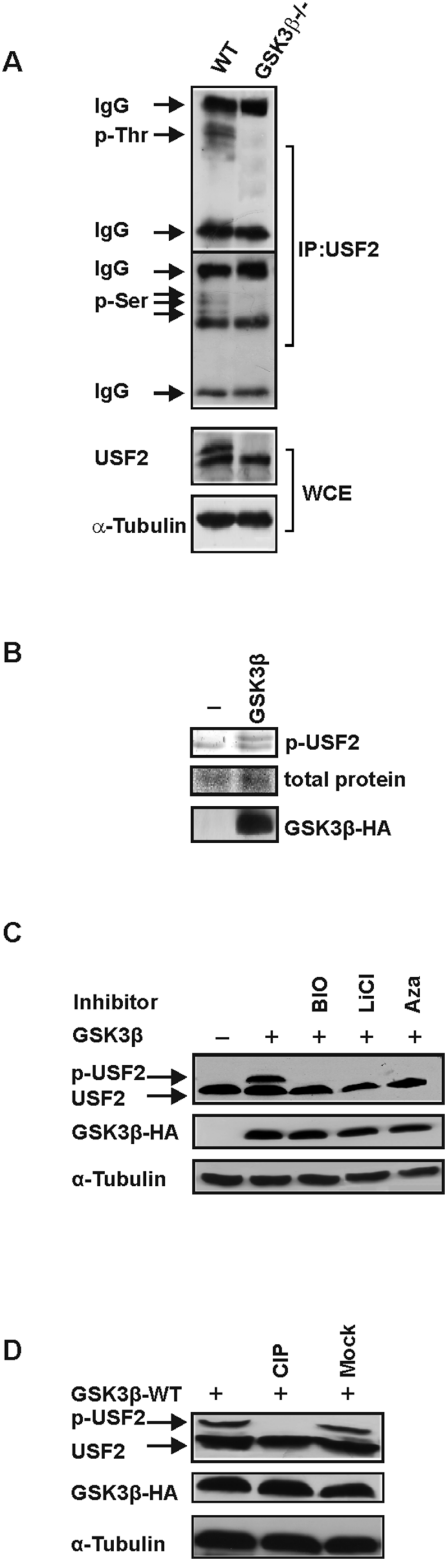
USF2 is phosphorylated by GSK3β. (A) USF2 was immunoprecipitated from GSK3β^+/+^ and GSK3β^−/−^ cells and phospho-USF2 protein levels were detected with phospho-threonine or phospho-serine antibodies. (B) USF2 was immunoprecipitated from HeLa cells transfected with pcDNA3-GSK3β-WT-HA. Following SDS-PAGE phosphoproteins were visualized with the Pro-Q Diamond Phosphoprotein Gel Stain. The total protein amount was detected by silver staining and GSK3β expression was verified by Western blotting. (C) Where indicated, HeLa cells transfected as above were treated with the GSK3 selective inhibitors BIO (1 µM), LiCl (10 mM), 1-Azakenpaullone (Aza, 7.5 µM) for 1 h. Proteins were isolated 24 h after transfection and detected by Western blotting. (D) Cells were transfected with expression vectors for USF2 and GSK3β and the cell extract was incubated with calf intestinal phosphatase (CIP) or only with buffer (Mock). Proteins were detected by Western blotting.

GSK3 has been found to phosphorylate many proteins among them transcription factors playing important roles in a variety of cellular processes [56]. Since we found that parts of the USF2 protein sequence match with the substrate recognition preferences of GSK3β [28] we aimed to investigate whether GSK3β can phosphorylate USF2 in intact cells and whether this affects USF2 function. To do this, we first immunoprecipitated USF2 from GSK3β-deficient mouse embryonic fibroblasts (GSK3β^−/−^ MEFs) and their wild-type counterparts (GSK3β^+/+^ MEFs) and visualized threonine and serine phosphorylated USF2 via immunodetection with phospho-threonine and phospho-serine antibodies. We found that threonine and serine phosphorylated USF2 could be detected in the wild-type cells whereas none of these forms could be detected in GSK3β^−/−^ MEFs (Fig. 1A). Next, we immunoprecipitated USF2 from HeLa cells transfected with a GSK3β expression vector and subsequently determined the phosphorylation of USF2 by phosphoprotein staining. We observed a positive phosphostaining in untreated HeLa cells indicating that some USF2 already exists in its phosphorylated form. In addition, a higher molecular weight and consequently slower migrating band indicating hyperphosphorylated USF2 could be detected upon overexpression of GSK3β (Fig. 1B).

We then used the molecular weight shift of phosphorylated USF2 to analyze phosphorylation of USF2 by Western blotting upon treatment of cells with 3 different kinase inhibitors supposed to be selective for GSK3β. All three kinase inhibitors, namely BIO, LiCl and 1-Azakenpaullone, prevented the appearance of phospho-USF2 (Fig. 1C). To further confirm that the band of higher molecular weight appearing after overexpression of GSK3β corresponds to phosphorylated USF2, cell extracts were incubated with calf intestinal phosphatase (CIP). After dephosphorylation with CIP, the band with the higher molecular weight disappeared, confirming that it indeed corresponds to the phospho-USF2 (Fig. 1D).

In addition to the experiments in HeLa cells, we were able to show that GSK3β-dependent phosphorylation of USF2 also occurs in HepG2 cells and in the human prostate cancer cell lines LNCaP and PC-3 (data not shown). Together, these data, in particular the data obtained from the GSK3β^−/−^ MEFs indicate that GSK3β phosphorylates USF2 in cells.

### Mapping of the GSK3β phosphorylation sites within USF2

**Figure 2:**
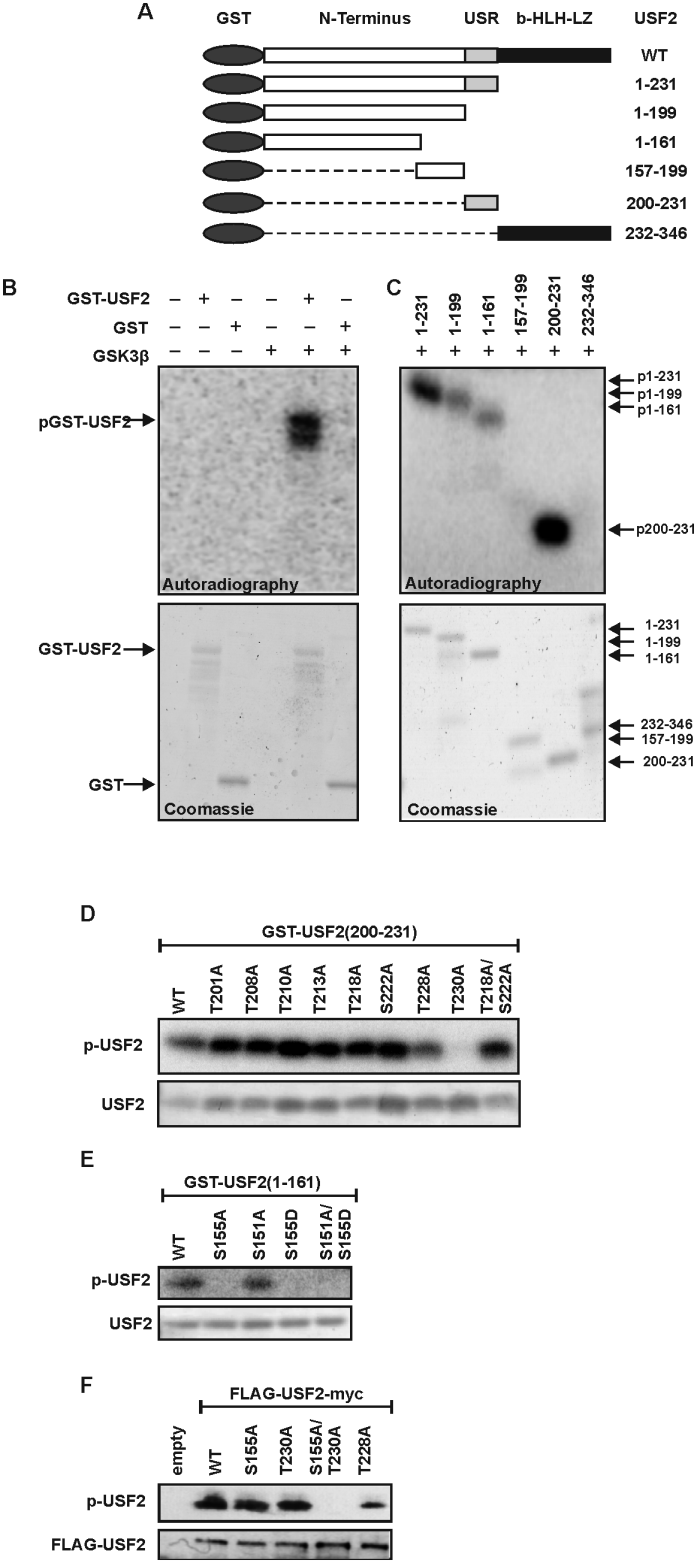
GSK3β-mediated phosphorylation occurs in two USF2 domains. (A) Schematic representation of the USF2 deletion mutants used to identify the domains that are phosphorylated by GSK3β. (B–E) Purified GST-tagged USF2 proteins were incubated with recombinant human GSK3β in the presence of [γ^−32^P] ATP. Proteins were separated by SDS-PAGE and incorporated radioactivity was detected by autoradiography. The total amount of proteins was detected by Coomassie staining. (F) HeLa cells were transfected with expression vectors for USF2 or the empty vector. USF2 was immunoprecipitated from the total cell extract and then incubated with recombinant human GSK3β in the presence of [γ^−32^P] ATP. Proteins were separated by SDS-PAGE and incorporated radioactivity was detected by autoradiography. The total amount of proteins was detected by silver staining.

To further investigate the details of GSK3β-dependent USF2 phosphorylation, we aimed to map the exact phosphorylation sites within USF2 by means of radioactive kinase assays with recombinant GST tagged USF2 proteins *in* *vitro*. In addition to full length USF2, we also purified 6 different deletion variants (Fig. 2A) to identify the domain within USF2 that can be phosphorylated by GSK3. In line with the previous data from the cells, we could confirm that GSK3 phosphorylates wild-type USF2 (Fig. 2B).

Next, we tested the 6 USF2 deletion mutants in kinase assays to determine which domains of USF2 can be phosphorylated by GSK3β (Fig. 2C). We detected a strong phosphorylation of the protein containing the USR (200–231) while a weaker phosphorylation occurred with the protein containing the N-terminal domain up to amino acid 161. No phosphorylation could be observed with the proteins containing the amino acids 157–199 or the b-HLH-LZ domain (232–346).

Based on these results we then aimed to identify the exact phosphorylation sites within these USF2 proteins. To do this, we first mutated all 8 phosphorylatable serine and threonine residues within the USR (200–231) separately and converted them into non-phosphorylatable alanine residues. Since the amino acids 218–222 (THPYS) within the USR perfectly match the GSK3β minimal recognition motif S/T-X-X-X-pS/pT, where the N-terminal S/T is the proper GSK3β phosphorylation site and the C-terminal S/T is the site of priming phosphorylation, we also generated a T218A/S222A double mutant. The kinase assays with these USR mutants and GSK3β revealed that T230 is the proper phosphorylation site within this domain, since replacement of this residue by alanine completely abolished the phosphorylation. All other USR single point mutants and the T218A/S222A double mutant were still phosphorylatable by GSK3β (Fig. 2D).

The N-terminal domain of USF2 comprising the amino acids 1–161 contains 18 phosphorylatable serine and threonine residues. We screened this sequence for minimal GSK3β recognition motifs and found one of these motifs within this N-terminal domain. Based on this, S151 would be the proper GSK3 phosphorylation site and S155 would be the site of priming phosphorylation. Hence, both sites were replaced by alanine. Additionally, S155 was substituted by aspartate; also a S151A/S155D double mutant was constructed. Kinase assays with these proteins revealed that any modification of S155 totally abolished GSK3β-mediated incorporation of radioactivity whereas the S151A mutant was still phosphorylatable. Since mutation of S155 completely prevented GSK3β-mediated phosphorylation, we concluded that this residue is the exclusive GSK3β site in this N-terminal domain of USF2 (Fig. 2E).

To verify the importance of S155 and T230 within full length USF2, we performed a kinase assay with recombinant GSK3β and full-length wild type USF2 or the respective S155 and T230 mutants which we expressed and immunoprecipitated from HeLa cells. The USF2-WT protein and the single mutants USF2-S155A, USF2-T230A, and as a control USF2-T228A were phosphorylatable by GSK3β. By contrast, when both S155 and T230 were substituted with alanine (S155A/T230A) phosphorylation by GSK3β was completely prevented (Fig. 2F).

Together, these data show that there are two residues within USF2, namely S155 and T230, which can be phosphorylated by GSK3β.

### Phosphorylation enhances the transactivity of USF2

**Figure 3:**
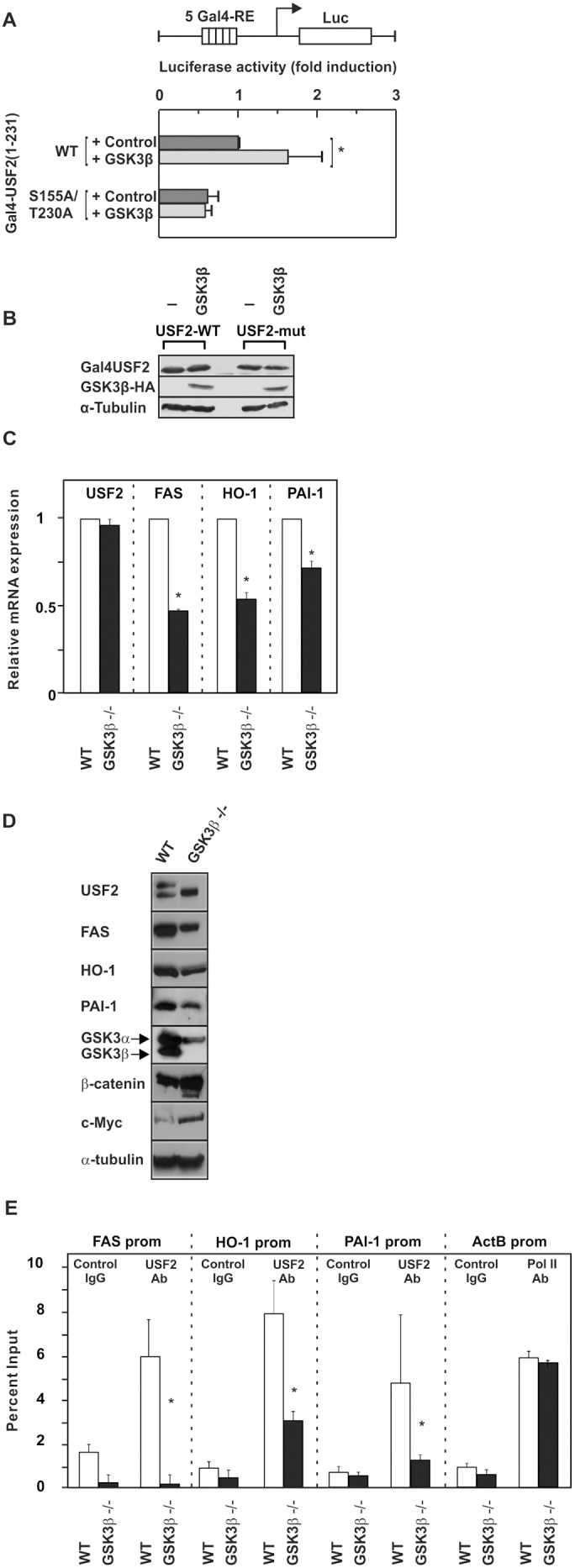
GSK3β-mediated phosphorylation of USF2 affects its transactivity, DNA binding and target gene expression. (A) HeLa cells were cotransfected with pFR-5Gal4-RE-Luc, an expression vector for constitutively active GSK3β-S9A and WT or mutant pcDNA6-Gal4-USF2 (1-231) or the appropriate empty Gal4 vector. The luciferase activity was calculated as fold induction compared to the Gal4-USF2 (1-231)-WT luciferase activity after subtracting the values from the empty Gal4 expression vector. *, significant differences control vs. GSK3β. (B) Representative Western blot of the transfected constructs. 50 µg of protein from transfected cells were probed with an antibody against Gal4, HA-tag and α-tubulin. (C) Quantitative RT-PCR analyses of FAS, HO-1, PAI-1 and USF2 mRNA levels in GSK3β^+/+^ and GSK3β^−/−^ cells. *, significant differences WT vs. GSK3β^−/−^. (D) Western Blot analyses of FAS, HO-1 and PAI-1 expression in GSK3β^+/+^ and GSK3β^−/−^ cells**.** 50 µg of protein were subjected to Western analysis with antibodies against FAS, HO-1, PAI-1 or β-catenin, c-Myc or α-tubulin; the latter served as a loading control. (E) ChIP was performed in GSK3β^+/+^ and GSK3β^−/−^ cells with either USF2 antibody, control IgG or RNA Pol II antibody. The quantitative PCR was performed with primers amplifying the FAS, HO-1, and PAI-1 promoter containing the USF2 binding sites, and with primers amplifying the β-actin promoter binding RNA Pol II as outlined in Materials and Methods. *, significant differences WT vs. GSK3β^−/−^.

To test whether the identified phosphorylation sites are crucial for the transactivity of USF2 we performed luciferase assays. We took advantage of the Gal4 system to assess the impact of the detected phosphorylation sites on the USF2 transactivity independent from their effect on the DNA binding capacity. Therefore, we generated constructs in which the USF2 DNA binding b-HLH-LZ domain (aa 232–346) was replaced with the DNA binding domain of the transcription factor Gal4. In addition to the resulting construct encoding the wild type Gal4-USF2 fusion protein (Gal4-USF2(1-231)-WT), another plasmid encoding a fusion protein in which the two GSK3β phosphorylation sites were substituted with alanine (Gal4-USF2(1-231)-S155A/T230A) was generated. As a control constructs encoding only the Gal4 DNA binding domain were used. These Gal4 fusion constructs were then cotransfected with a plasmid containing the luciferase gene under the control of 5 Gal4 response elements (RE). Thus, changes in the measured luciferase activity reflect the transactivity of the transcription factor USF2. While the presence of the constitutively active GSK3β-S9A significantly increased the observed luciferase activity with the Gal4-USF2(1-231)-WT construct, no change in luciferase activity could be detected with the Gal4-USF2(1-231)-S155A/T230A double mutant (Fig. 3A). Western Blot controls revealed that all Gal4-USF2 proteins used in the luciferase experiments were correctly expressed and their levels were not affected by overexpression of GSK3β (Fig. 3B). Together, these data strongly suggest that phosphorylation of USF2 by GSK3β is critical for the regulation of USF2 transactivity.

### Phosphorylation of USF2 increases expression of its target genes

Next, we wanted to know whether the phosphorylation of USF2 affects expression of the USF2 target genes heme oxygenase 1 (HO-1), plasminogen activator inhibitor 1 (PAI-1), and fatty acid synthase (FAS). To test this, we used GSK3β^+/+^ and GSK3β^−/−^ cells and measured FAS, HO-1, and PAI-1 mRNA and protein levels. Indeed, FAS, HO-1, and PAI-1 mRNA levels (Fig. 3C) as well as protein levels (Fig. 3D) were reduced significantly in the GSK3β^−/−^ cells. By contrast, non-USF2 targets but GSK3β regulated proteins like β-catenin and c-Myc were upregulated in GSK3β^−/−^ cells (Fig. 3D). Thus, these data indicate that phosphorylation of USF2 by GSK3β enhances expression of USF2 target genes.

### Phosphorylation of USF2 increases its DNA binding to target gene promoters

Since S155 and T230 are close to the DNA binding domain of USF2, we also decided to investigate whether the phosphorylation by GSK3β affects binding of USF2 to its target DNA. To do this, we used again GSK3β^+/+^ and GSK3β^−/−^ cells and performed chromatin immunoprecipitations with an USF2 antibody followed by quantitative PCR with primers encompassing the USF binding sites within the FAS, HO-1, and PAI-1 promoter [21]. The chromatin immunoprecipitation studies show that USF2 strongly binds to the FAS, HO-1, and PAI-1 promoters in GSK3β^+/+^ cells. By contrast, binding of USF2 to the FAS promoter was almost lost in **GSK3β^−/−^** cells and binding to the HO-1 and PAI-1 promoter was reduced by 60% and 70%, respectively (Fig. 3E). Furthermore, binding of RNA polymerase II to the β-actin promoter, used as a positive control, showed no significant difference between WT- and GSK3β^−/−^ cell lines (Fig. 3E). Together, these data indicate that phosphorylation of USF2 by GSK3β contributes to the regulation of its DNA binding capacity.

**Figure 4:**
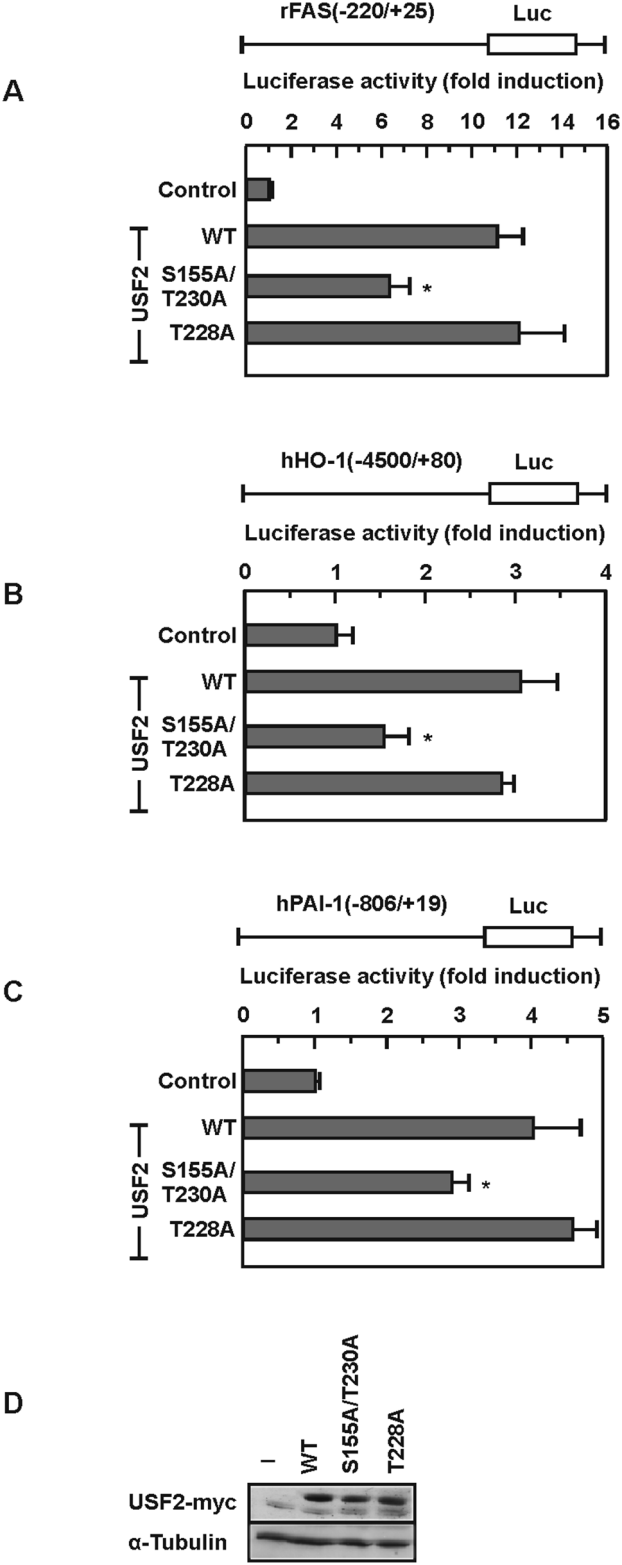
GSK3β-mediated phosphorylation of USF2 affects activation of target gene promoters. (A–C) HeLa cells were cotransfected with the indicated luciferase gene construct and with WT or mutant p3xFLAG-USF2-myc-CMV24 or the appropriate empty vector. The measured luciferase activity is plotted as fold induction compared to the luciferase activity measured in the control transfected with the empty expression vector. *, significant difference WT vs. mutant. (D) Representative Western blot of the transfected constructs. 50 µg of protein from transfected cells were probed with an antibody against myc-tag and α-tubulin.

To address whether the reduced DNA binding and transactivation capacity has direct transcriptional consequences, we used FAS, HO-1, and PAI-1 promoter luciferase constructs and cotransfected them with vectors for wild-type USF2 (USF2–WT), USF2-S155A, USF2-T230A or the S155A/T230A double mutant. As a control, the promoter luciferase constructs were cotransfected with a USF2 plasmid in which T228 was substituted with alanine (Fig. 4). The luciferase assays revealed that wild-type USF2 induced luciferase activity significantly with all tested promoters. By contrast, the USF2-S155A and USF2-T230A single mutants and the USF2-S155A/T230A double mutant diminished luciferase activity compared to USF2-WT with all tested promoter constructs. The control mutant USF2-T228A induced luciferase activities like USF2-WT (Fig. 4). Together, these data indicate that phosphorylation of USF2 by GSK3β at S155 and T230 are important for the regulation of USF2 DNA binding and transactivity.

### Phosphorylation changes the 3-dimensional structure of USF2

**Figure 5:**
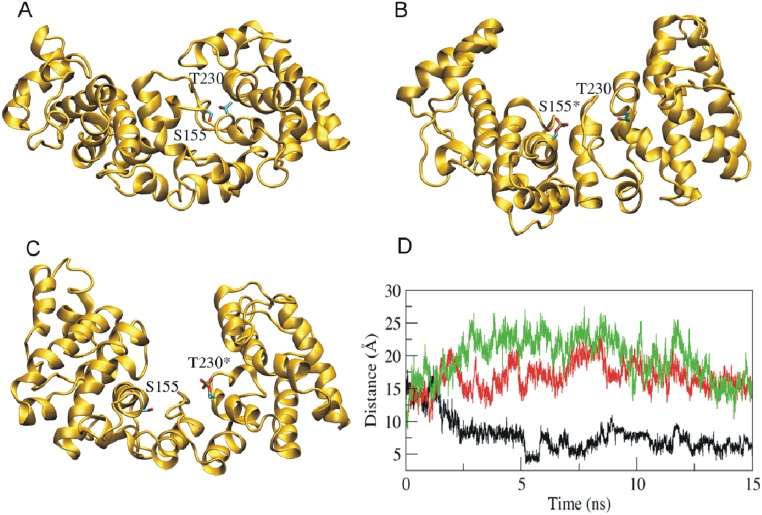
Phosphorylation of USF2 by GSK3β increases domain (residue) distance. (A–C) Simulated structures of wild type (A), S155 phosphorylated (B), and T230 phosphorylated (C) USF2 from the MD simulation trajectories. The side chains of Ser155 and Thr230 are shown in stick representation. Phosphorylated amino acids are labeled with an asterisk (*). The distance between side chain oxygen of S155 and T230 were analyzed (D). Phosphorylation of S155 (red) or T230 (green) increases the distance compared to non-phosphorylated USF-2 (black).

Next we aimed to understand whether the phosphorylation of USF2 at S155 or T230 affects the 3-dimensional structure of the protein. Since there is no crystal structure for full length USF2 available we performed a series of molecular dynamics simulations of USF2 when S155 and T230 remained non-phosphorylated (native protein) and when S155 and T230 were phosphorylated. The modeled molecular structure of the native non-phosphorylated USF2 protein indicated that the protein is composed of two domains (Fig. 5A). The two GSK3β phosphorylation sites S155 and T230 are located at the interface of the two domains. The respective 15 ns molecular dynamics simulations with phospho-S155 and phospho-T230 were sufficient long for a proper stabilization of the C-alpha root mean square deviations (data not shown). We found that the distance between the two domains increased (Fig. 5B–D) in phosphorylated USF2. In particular, the distance between side chain oxygen atoms of S155 and T230 residues were increased by 10 Å (Fig. 5). Together, the results show that phosphorylation of USF2 by GSK3β at S155 and T230 effectively increases the separation of the two major USF2 domains suggesting that this contributes to the changed DNA binding and transactivity.

### GSK3β- affects the half-life of USF2

**Figure 6:**
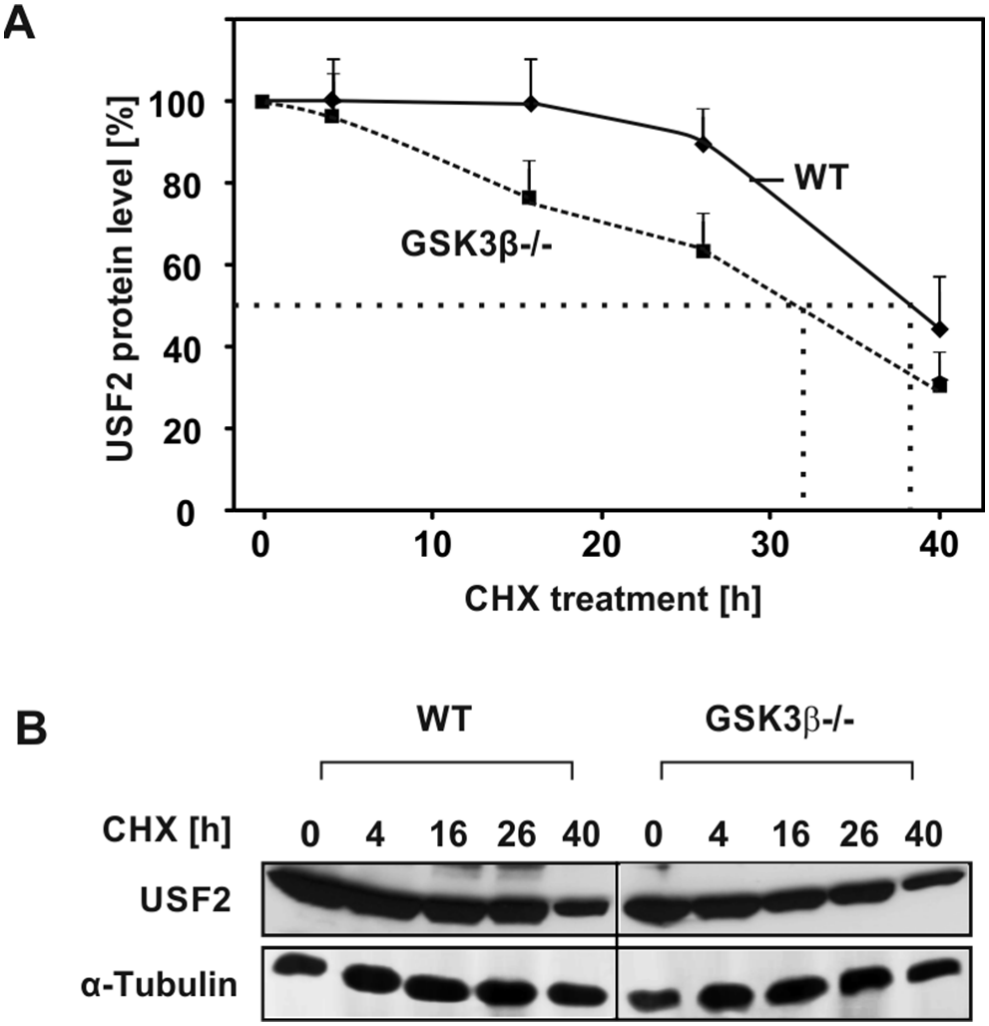
The half-life of USF2 is induced upon GSK3β-dependent phosphorylation. (A) GSK3β^+/+^ and GSK3β^−/−^ cells were treated with 10 µg/ml CHX for the indicated time periods. Proteins were isolated, separated by SDS-PAGE and detected by Western blotting. Protein levels were quantified and the relative protein level of USF2 was blotted against the duration of CHX treatment for estimation of the half-life. The dashed line indicates the USF2 half-life where 50% of the USF2 protein level was reached. (B) Representative Western Blot. 50 µg of protein from were probed with an antibody against USF2 and α-tubulin.

Often phosphorylation or dephosphorylation changes the stability of a protein. Hence, we investigated whether GSK3β-dependent phosphorylation affects USF2 protein stability. Therefore, GSK3β^+/+^ and GSK3β^−/−^ cells were treated with the protein synthesis inhibitor CHX and the level of endogenous USF2 was monitored by Western blotting. While the USF2 half-life in the control cells was about 38 h, the half-life in cells lacking GSK3β was estimated to be about 31 h (Fig. 6). These data show that GSK3β-mediated phosphorylation increases the protein stability of USF2.

### The phosphorylation of USF2 by GSK3β does not affect cell proliferation but influences cell migration

**Figure 7:**
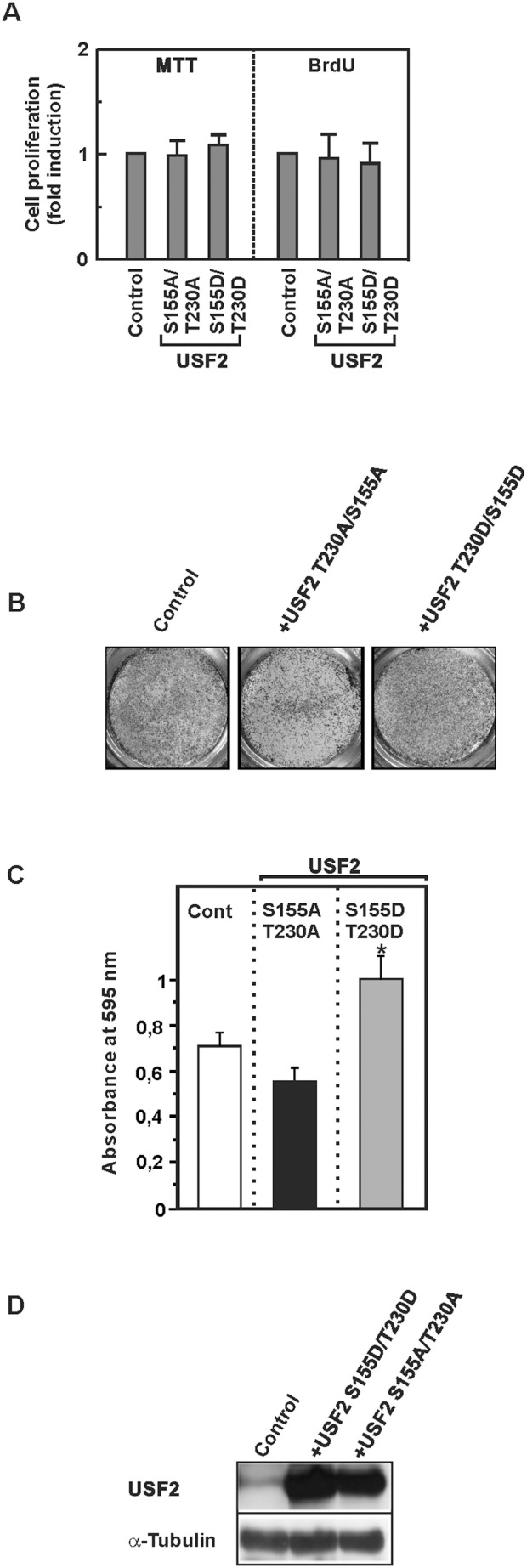
The phosphorylation of USF2 by GSK3β affects cell migration. (A, B, C, D) GSK3β^−/−^ cells were transfected with vectors allowing expression of USF2-S155A/T230A or USF2-S155D/T230D or an empty vector. Cellular viability, proliferation and cell migration were monitored by MTT, BrdU (A) and cell migration (B, C) assays. (B) Photographs from a representative Transwell chamber experiment. (C) Data represent the absorbance of crystal violet at 595 nm relative to the control. *, significant difference between GSK3β^−/−^ cells and GSK3β^−/−^ cells + USF2-S155D/T230D. (D) The expression of USF2 was controlled by Western blotting. 50 µg of protein from transfected cells were probed with an antibody against USF2 and α-tubulin.

We next determined whether the GSK3β mediated USF2 regulation is involved in cell proliferation. To test this, we used GSK3β^−/−^ cells and transfected them with the non-phosphorylatable USF2 variant S155A/T230A or the USF2 variant S155D/T230D mimicking a GSK3 phosphorylated status and measured the overall cellular viability and metabolic activity of the cells in an MTT assay as well as bromodesoxyuridine (BrdU) incorporation into newly synthesized DNA. We found that none of the USF2 variants exerted an effect on cellular viability and DNA synthesis (Fig. 7A).

We also analyzed cellular viability and BrdU incorporation in HeLa cells expressing the non-phosphorylatable USF2 -S155A/T230A or the USF2 variant S155D/T230D mimicking a GSK3 phosphorylated status along with GSK3β. Again, it turned out that neither the non-phosphorylatable variant USF2-S155A/T230A nor the phospho mimicking USF2 variant S155D/T230D had an effect on cellular viability or BrdU incorporation (data not shown). These data suggest that phosphorylation of USF2 by GSK3β has no impact on overall cellular viability and proliferation.

To analyze whether the GSK3β-mediated effects on USF2 have an impact on the invasive cellular potential, we performed transwell migration assays with GSK3β^−/−^ cells and GSK3β^−/−^ cells transfected with either the non-phosphorylatable USF2 variant S155A/T230A or the phospho-mimicking variant S155D/T230D. A significant increase in migration could be detected with GSK3β^−/−^ cells expressing the phospho-mimicking variant S155D/T230D; which reflects GSK3 phosphorylated USF2 whereas the USF2 S155A/T230A variant slightly decreased migration (Fig. 7B, C, D). Together, these data show that lack of GSK3β can reduce invasive cellular growth in a USF2-dependent manner.

## Discussion

In the current study we investigated the impact of GSK3β on the phosphorylation and function of the transcription factor USF2. Our data demonstrated several new findings with respect to USF2 phosphorylation and regulation. First, it was found that GSK3β phosphorylates USF2 at S155 and T230. Second, phosphorylation of USF2 at S155 and T230 increased its DNA binding, transactivity, half-live, and target gene expression. Third, phosphorylation of USF2 by GSK3β did not affect cell proliferation but increased cell migration.

From the two USFs known, mainly USF2 appeared to have a direct link to cell migration and thus to cancerogenesis. This was first highlighted by a study investigating hormone refractory prostate cancer samples. Half of the hormone refractory prostate cancer samples displayed a loss of chromosome 19q ter-q13.1 [57] which includes the region with the *usf2* gene [58]. A reintroduction of an intact human chromosome 19 into a tumorigenic prostate cell line reduced tumorigenicity in athymic nude mice [59]. In addition, USF2 was found to be affected in breast [9], lung [11], oral [10] and colorectal [60] cancer suggesting a more general role of USF2 in tumorigenesis. This suggestion is supported by reports showing that USF2 also regulates the expression of the APC, BRCA2 and p53 tumor suppressor genes [61]–[63] and our previous studies showing that USF2 plays an important role in regulating the expression of the cancer marker PAI-1 [21], [64].

Although these observations suggest that USF2 plays an important role during cancerogenesis, it was almost unknown which mechanisms are responsible for the regulation of the transcription factor. The current study narrows this gap and is the first to report that phosphorylation of USF2 by GSK3β affects DNA binding, transactivation and protein half-life. These data are in line with previous reports from other groups and our own work indicating that the effect of USF2 on cancerogenesis may be due to post-translational modifications like phosphorylation. Although no mechanism was determined, some reports indicated that the USF-specific region (USR) appears to be crucial for the function of USF since the simple use of USF2 variants consisting just of the leucine zipper (LZ), basic helix-loop-helix (bHLH) and basic region (BR) which are involved in dimerization, DNA binding and nuclear localization [65] or the N-terminally located transactivation domain (TAD) had no important effect on colony formation [66] or PAI-1 expression [21], [64].

Interestingly, when inspecting the USF2 sequence for the presence of GSK3 sites, we found the existence of some S/T-X-X-X-S/T motifs, which are typical recognition motifs for GSK3β. Although not strictly required, priming phosphorylation seems to increase substrate phosphorylation by GSK3β [18]. Within the minimal GSK3 recognition motif, the first S/T is the proper GSK3β target site, X is any amino acid and the C-terminal S/T residue is the site of priming phosphorylation [28]. The USR sequence 218-THPYS-222 completely matches this consensus sequence. However, the kinase assays and transfection experiments with USF2 variants mutated at the putative GSK3 sites T218 and S222 did not show that these residues are real GSK3 sites. Instead, T230 was determined to be the site of USR phosphorylation. Similarly, analyzing the USF2 motif 151-SNGGS-155, we found that instead of the theoretical phosphorylation site S151 the real site of GSK3β-dependent phosphorylation is S155. A recent study on the USF1 protein which is a product from a different gene but can heterodimerize with USF2 identified Thr153 and S186 to be GSK3 sites [18]. Although some similarities between USF1 and USF2 exist, they appear to have different functions, and different target genes which is also substantiated by significantly different phenotypes of USF1 and USF2 knockout mice. Moreover, like in the present study there is also no priming phosphorylation at the amino acid in +4 position of USF1 S186 [18]. These findings are conform to other studies in which for example the transcription factors c-Jun [67], p53 [68] and c-Myb [69] were found to be phosphorylated by GSK3β at sites not matching the minimal recognition motif. Checking the USF2 sequence adjacent to the two identified phosphorylation sites S155 and T230 more closely revealed that the n+4 position, which is the site of priming phosphorylation in the preferred GSK3β recognition motif, is a glutamate (E) residue in both cases (155-SPAAE, 230-TPRDE). Considering that glutamate has a negative charge like a phosphate group, it is possible that the glutamate residue can mimic priming phosphorylation and thereby favors GSK3β-mediated phosphorylation of the two identified USF2 sites.

In addition to our own experiments, the only data for USF2 phosphorylation so far came from a study of the bovine system investigating the effect of forskolin and protein kinase A (PKA) on the prostaglandin G/H-2 synthase promoter. That study showed that USF2 contains three PKA phosphorylation sites and that overexpression of PKA enhanced USF DNA binding in bovine granulosa cells. Interestingly, mutations of the putative PKA phosphorylation sites S259, S269 and S275 in USF2 reduced but did not completely abolish transactivation capacity [17]. These findings are nicely complemented by the findings of our study showing that not only PKA but also GSK3 is of importance for the regulation of USF2 DNA binding activity and transactivity. Like in the previous report, our study also showed that mutation of the GSK3 sites in USF2 did not completely abolish its transactivity. This can be explained by the fact that the presence of the transactivation domain alone accounts for a large extend of basal transactivity and that mutation of the phospho-sites only affects the additional increase normally mediated by post-translational phosphorylation. In addition, this view is supported by our data from the structural simulation experiments where the phosphorylation of USF2 by GSK3 only shifts the distance between the two major structural domains but does not alter its overall conformation.

It already has been shown that the protein stability of several proteins which play a role in cancerogenesis is altered upon GSK3β-dependent phosphorylation, among them are for example the transcription factors HIF-1α [70], [71], c-Jun [67], p53 [68] and c-Myb [69], c-Myc [70] and β-catenin [72]. Our study is in line with these findings since cMyc and β-catenin protein levels were found to be enhanced in the GSK3β lacking MEFs (Fig. 3C). In addition, our findings allow adding USF2 to this list of these proteins since our half-life studies revealed that lack of GSK3β-mediated phosphorylation reduces the half-life of USF2. Future experiments need to unravel whether modifications in ubiquitination followed by proteasomal degradation or other mechanisms contribute to this phenomenon.

The induction of USF2 transactivity due to phosphorylation by GSK3β is complemented by the induction in USF2 DNA binding capacity and half-life. At the same time an increased expression of the USF2 target genes FAS, HO-1, and PAI-1 is initiated. This type of regulation, together with the findings of the migration assay is in line with previous reports indicating that GSK3β and USF2 have a role in cancerogenesis [8], [73].

However, the exact function of both, GSK3β and USF2 in cancerogenesis appears to be variable and may depend on the cellular context. While a number of studies support the idea of GSK3β being tumor suppressive, others studies showed that GSK3β may promote cancer development [72]. Similar findings were obtained with respect to USF2; being tumor suppressive with respect to prostate cancer [8], [73] but being rather promoting in the development of lung cancer [74] and thyroid cancer [75]. Although no study has yet correlated the activity of GSK3β with the activity of USF2 in a certain tumor setting, the findings of the present study would favor the tumor promoting aspects of GSK3β and USF2 since GSK3β activated USF2 enhanced cell migration which may be important in terms of tumor cell metastasis. In addition, cross-talk(s) between signalling pathways depending on different growth conditions, the cellular context and/or tissue-specific aspects may well be other player(s) influencing the activity of USF2.

In summary, our study identified GSK3β as a kinase that phosphorylates USF2 and thereby regulates its function. The GSK3β-mediated phosphorylation of USF2 at S155 and at T230 leads to increased transactivity, DNA-binding, and half-life of the transcription factor and to an induction of cell migration. These novel findings on the regulation of the transcription factor USF2 set the basis for further studies investigating how these mechanisms contribute to the development of different types of cancer.
